# The association between receipt of home care rehabilitation services and acute care hospital utilization in clients with multimorbidity following an acute care unit discharge: a retrospective cohort study

**DOI:** 10.1186/s12913-023-09116-0

**Published:** 2023-03-18

**Authors:** Amanda Mofina, Jordan Miller, Joan Tranmer, Wenbin Li, Catherine Donnelly

**Affiliations:** 1 School of Public Health Sciences, University of Waterloo, Waterloo, ON Canada; 2 School of Rehabilitation Therapy, Queen’s University, Kingston, ON Canada; 3 School of Nursing, Queen’s University, Kingston, ON Canada; 4 ICES, Queen’s, Kingston, ON Canada

**Keywords:** Occupational therapy, Physical therapy, Rehabilitation, Multimorbidity, Home care

## Abstract

**Background:**

Individuals experiencing multimorbidity have more complex healthcare needs, use more healthcare services, and access multiple service providers across the healthcare continuum. They also experience higher rates of functional decline. Rehabilitation therapists are well positioned to address these functional needs; however, little is known about the influence of rehabilitation therapy on patient outcomes, and subsequent unplanned healthcare utilization for people with multimorbidity. The aims of this study were to: 1) describe and compare the characteristics of people with multimorbidity receiving: home care rehabilitation therapy alone, other home care services without rehabilitation therapy, and the combination of home care rehabilitation therapy and other home care services, and 2) determine the association between home care rehabilitation therapy and subsequent healthcare utilization among those recently discharged from an acute care unit.

**Methods:**

This retrospective cohort study used linked health administrative data housed within ICES, Ontario, Canada. The cohort included long-stay home care clients experiencing multimorbidity who were discharged from acute care settings between 2007–2015 (*N* = 43,145). Descriptive statistics, ANOVA’s, t-tests, and chi-square analyses were used to describe and compare cohort characteristics. Multivariable logistic regression was used to understand the association between receipt of rehabilitation therapy and healthcare utilization.

**Results:**

Of those with multimorbidity receiving long-stay home care services, 45.5% had five or more chronic conditions and 46.3% required some assistance with ADLs. Compared to people receiving other home care services, those receiving home care rehabilitation therapy only were less likely to be readmitted to the hospital (OR = 0.78; 95% CI: 0.73–0.83) and use emergency department services (OR = 0.73; 95% CI: 0.69–0.78) within the first 3-months following hospital discharge.

**Conclusions:**

Receipt of rehabilitation therapy was associated with less unplanned healthcare service use when transitioning from hospital to home among persons with multimorbidity. These findings suggest rehabilitation therapy may help to reduce the healthcare burden for individuals and health systems. Future research should evaluate the potential cost savings and health outcomes associated with providing rehabilitation therapy services for people with multimorbidity.

## Introduction

The prevalence of multimorbidity, the co-occurrence of two or more chronic conditions, is estimated at upwards of 33.1% globally [1]. Individuals experiencing multimorbidity are more likely to be admitted to hospital compared to those without multimorbidity [2]. Those with four or more physical chronic health conditions are nearly six times more likely to experience an unplanned hospital admission [2]. It is evident that individuals who experience multimorbidity interact with, and transition through, the health care system more frequently because they have higher health care needs that span across multiple health domains [2–7].

Interprofessional healthcare teams support individuals with complex health needs to navigate health care systems, particularly the transitions between systems [8]. Rehabilitation therapists (occupational therapists and physical therapists) are members of interprofessional healthcare teams that focus on improving patient function by considering multiple aspects of health such as physical, psychosocial, cognitive, addressing the person’s abilities as well as their environment, and social determinants of health [9–13]. As such, they are well positioned to address the complex functional needs of persons with multimorbidity.

There is a dearth of evidence examining the impact of rehabilitation therapy for individuals with multimorbidity, and the subsequent impact on healthcare utilization. A recent rapid review explored the relationship between home care rehabilitation, functional outcomes, and subsequent health utilization for those experiencing multimorbidity and found just four studies [12]. A retrospective cohort study (*N*= 99,764 home care clients) included within the rapid review reported that rehabilitation therapists can contribute to a reduction in hospital readmissions and institutionalization (long-term care admission) for people with musculoskeletal health conditions [11]. However, there was a gap in the literature with respect to understanding the association between receipt of home care rehabilitation therapy and subsequent health utilization following a discharge from an acute inpatient hospital unit among those with multimorbidity.

This study aims to address this gap in the literature through the following objectives: 1) To describe and compare the characteristics of people with multimorbidity who are referred and receiving home care rehabilitation therapy to those receiving home care for other services after recent discharge from an acute care unit in Ontario, and 2) to identify the association between home care rehabilitation therapy and subsequent health utilization (hospital readmission and emergency department use) by people with multimorbidity recently discharged home from an acute care unit in Ontario. Addressing these research gaps will build upon existing literature by determining the role of rehabilitation therapists in reducing unplanned healthcare use after transitions out of the hospital for people with multimorbidity.

## Methods

### Study design and setting

This retrospective cohort study used linked health administrative data in Ontario, Canada between the years 2007–2015. This time range was selected because it corresponds with an eight-year period of structural stability in the home care delivery model in the province. This timeframe corresponds with the co-existence of Local Health Integration Networks (LHINs) and Community Care Access Centres (CCACs). The LHINs and CCACs were responsible for home care service funding, eligibility, and access in Ontario.

### Data sources

Health administrative data for Ontario residents are housed at ICES, a not-for-profit organization that aims to improve health care using existing data to further the evidence. ICES is a prescribed entity operating under data security policies and procedures approved by the Ontario Information and Privacy Commissioner. Multiple datasets housed within ICES were used and these datasets were linked using unique encoded identifiers and analyzed at ICES. The Registered Persons Database (RPDB) includes data related to population demographic characteristics. The Discharge Abstract Database (DAD) includes data on hospital discharges and the National Ambulatory Care Reporting System (NACRS) includes data regarding emergency department utilization. The Resident Assessment Instrument-Home Care database (RAI-HC database) was used to provide details about home care services received and key measures of functional status.

Additional databases were used to identify individuals with multimorbidity, which will be further outlined below.

### Datasets used in defining the multimorbidity population

An established ICES macro was used to identify individuals with multimorbidity for this analysis. Multimorbidity was defined as experiencing two or more co-occurring chronic conditions and was considered in the context of seventeen chronic conditions. The ICES cohort included the following chronic conditions based on prevalence and system-level burden: **acute myocardial infarction (AMI)**, osteoarthritis and other arthritis (excluding rheumatoid arthritis), **rheumatoid arthritis, asthma**, all cancers, cardiac arrythmia, **congestive heart failure, chronic obstructive pulmonary disease**, coronary syndrome (excluding AMI), **dementia, diabetes, hypertension**, mood disorders (anxiety, depression and other nonpsychotic disorders), other mental illnesses, osteoporosis, renal failure, and stroke (excluding transient ischemic attacks) [7, 14–21]. The ICES derived chronic condition cohorts have been validated for eight of the 17 chronic conditions considered in the multimorbidity definition (bolded in the above list) [22–27]. The other nine conditions were defined using similar methods to the validated ICES chronic condition cohorts [22].

### Client population

Individuals were included in the cohort if they were: 1) diagnosed with multimorbidity as defined above, 2) were discharged home from the acute care unit, 3) long-stay home care clients, which refers to those who are expected to receive home care services for a minimum of 60 days [11], and had one RAI-HC assessment within 15 days from their hospital discharge, which is the index event (excluding home care discharge assessments), and 4) above the age of 18 and less than 105 years of age. The lookback window for capturing the chronic conditions used in the definition of multimorbidity was five years prior to the index date (the individuals’ first home care assessment following hospital discharge). The RAI-HC assessment tool is a validated standardized, mandated assessment completed with all long-stay home care clients in Ontario [28]. This assessment tool captures demographic information as well as aspects of cognitive health, psychoemotional health, physical functioning and mobility, and other domains of health. This tool also has embedded health subscales that capture some of these larger functional constructs, which include: Activities of Daily Living (ADL) Hierarchy Scale, Instrumental Activities of Daily Living (IADL), Pain Scale, Cognitive Performance Scale (CPS), Depression Rating Scale (DRS) and Changes in Health, End-stage Disease, Signs and Symptoms Scale (CHESS) used to further describe functional and health statuses [28–32]. The proximity of the RAI-HC assessment with the hospital discharge (within 15 days) was an important consideration in the transition from hospital care to home care because of the relationship being explored in the current study: the relationship between receipt of home care rehabilitation services and subsequent unplanned healthcare service utilization. Furthermore, this 15-day time-period aimed to exclude those with rapid readmissions who would not have been home long enough to have home care services initiated and/or implemented.

**Fig. 1:**
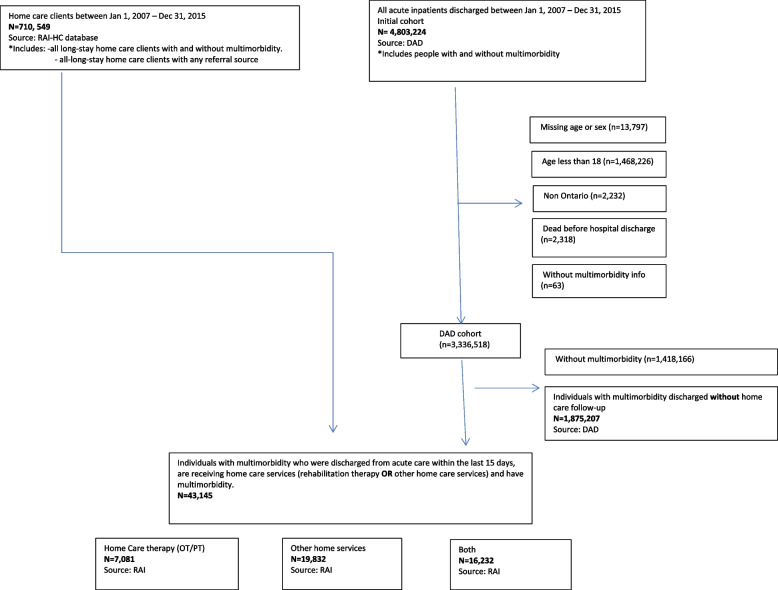
Study population flowchart

Exclusion criteria for the cohort included individuals: 1) with an invalid unique ICES identifier, 2) with an invalid code for age and/or sex, 3) who died at the hospital, or their date of death preceded the receipt of home care services, 4) who were non-Ontario residents, 5) resided in an institutionalized care environment and/or were discharged to an institutionalized environment (i.e., long-term care or hospital residence). The two cohorts, the home care cohort derived from the RAI-HC database and the acute care cohort derived from the DAD were then linked to create the study cohort of home care clients with multimorbidity who were discharged from an acute care unit. Figure 1 illustrates the cohort creation process. Of note, the individuals removed from the acute care discharges could populate more than one exclusion group; that is, these exclusion groups were not mutually exclusive at this stage. For example, one individual could be included in the missing age or sex, and age less than 18 groups and counted twice. The ‘DAD cohort’ however, is the calculated difference with individuals counted only once.

### Variables

Home care referrals in Ontario can be made by a healthcare provider, a caregiver, and/or a self-referral. These referrals can occur at any point along the continuum of care and eligibility is determined by a case manager [33, 34]. For this study, the individuals were categorized into one of the following mutually exclusive groups using the treatment variable based on the home care that they received after their recent discharge from an acute care hospital (within 15 days). The groups were: 1) rehabilitation therapy only (occupational therapy and/or physical therapy); 2) rehabilitation therapy and other home care services; 3) other home care services excluding those receiving occupational therapy and/or physical therapy. The other home care services could include services such as, but not limited to, home care nursing, personal support work, and social work.

The outcomes of interest were unplanned hospital admission and emergency department visits after long-stay home care rehabilitation services. The three treatment groups were compared with respect to the outcome of interest. Hospital admissions and emergency department visits were dichotomized (yes/no) and captured at 3 months and 12 months from the time of an individuals’ first RAI-HC assessment post-initial hospital discharge. That is, if an individual was readmitted at any point within three months, they would be coded as having experienced an admission; similarly, if an individual was readmitted at any point within 12-month observation window, they were coded as experiencing an admission. When conducting the supplemental analysis, the sum of the readmissions at the three-month time frame and the sum of the readmissions for the 12-month timeframe were considered. The same coding structure was applied for emergency department utilization.

The RAI-HC subscales, along with other demographic characteristics derived from the assessment were used as potential covariates in the analysis (e.g., age, cognition, and functional performance in areas of activities of daily living and instrumental activities of daily living).

### Analysis

Descriptive statistics were used to summarize the characteristics of people with multimorbidity who were discharged from an acute care setting who received home care and those that did not. Descriptive statistics were also used to describe the characteristics of people with multimorbidity who received home care rehabilitation, those who received other home care services, and those that received rehabilitation and other home care services. T-tests and ANOVAs were used to compare the means across continuous variables and chi-square tests were used to compare categorical variables among baseline characteristics of people who received different types of home care. The primary analysis involved multivariable logistic regression to determine the relationship between home care rehabilitation therapy and subsequent hospital utilization. The initial step in building the model(s) included the development of an a priori list of potential covariates informed by the literature, to consider when examining the relationship between receipt of home care rehabilitation and subsequent health utilization (e.g., age, sex, number of chronic conditions, cognition, areas of functional performance, and indicators of marginalization) [10, 14, 19, 35–37]. These covariates aligned with the clinical and social characteristics discussed in the theoretical model proposed by Rogers et al. [10] that was foundational in guiding this research. Secondly, univariate logistic regression models were used to inform covariate selection. Covariates that were retained included those that were significant in the descriptive analysis and had statistically significant univariate relationship with hospital re-admissions and emergency department visits. These remaining covariates were entered into backwards elimination stepwise regression procedure. A decision was made to consider the covariates across two models to ensure methodological consistency. The models included: 1) covariates retained in the backwards elimination model, and 2) non-modifiable covariates only (age, sex, and number of chronic conditions).

Additionally, to assess the robustness of the findings, supplementary analysis involved examining the outcome, healthcare utilization, as a count variable over the 3-month and 12-month timeframe. Negative binomial regression was used to consider this relationship because it includes a dispersion term that corrects for a high number of ‘0’ values [38, 39]. In this study, ‘0’ values refer to no hospital admission or no emergency department interaction. This approach ensures a more accurate variance estimate [38, 39]. These models considered only non-modifiable covariates because there was less than 10% difference between the above two multivariable logistic regression models.

This study was approved by the Queen’s University Health Sciences and Affiliated Teaching Hospitals Research Ethics Board in Kingston, Ontario, Canada (approval #6,025,299-REH-739–18). All statistical analyses were conducted using SAS Enterprise guide, version 7.1 (SAS Institute, Cary, NC).

## Results

There were 4,803,224 acute care inpatients discharged between January 2007 and December 2015. Of those, 1,875,207 had multimorbidity and were discharged without home care services. In the same timeframe, 710,549 individuals received long-stay home care services and of those 43,145 individuals had a diagnosis of multimorbidity, received long-stay home care services and were captured within a discharge timeframe of 15 days. Individuals who returned to the hospital within the 15-day timeframe and short-stay home care clients were not captured in this study. Of those receiving home care included in the study cohort, 54% received home care rehabilitation therapy (occupational therapy, or physiotherapy) services.

Those who did not receive home care had an average age at discharge of 62.5 (sd = 17.0) years, a higher proportion were female (54.3%), and the majority had two or three chronic conditions (57.6%). Most clients receiving home care were over the age of 75 years (67.6%), and female (62%).

Of the clients receiving rehabilitation therapy services only, a higher proportion experienced mild, moderate, or severe cognitive impairment (58.2% vs 47.3%) and required supervision or assistance with activities of daily living (49.7% vs. 39.9%) compared to those not receiving rehabilitation therapy (*p* < 0.001) (Table 1). A higher proportion of those receiving rehabilitation therapy services had experienced falls compared to those receiving other home care services only (51.6% of those receiving rehabilitation therapy only, 46.6% of those receiving rehabilitation therapy and other home care services, and 35.7% of those receiving other home care services only; *p* < 0.001) (Table 1).

**Table 1:** Distribution of baseline characteristics and embedded health subscales across home care health professions

Variable	Other Home care Services only *n* = 19,832 n(%)	Home care rehabilitation therapy only *n* = 7,081 n(%)	Home care rehabilitation therapy and other services *n* = 16,232 n(%)	Total *N* = 43,145 n(%)	*P* value
**Age**					
**Mean (SD)**	76.6 (12.2)	78.5 (11.8)	77.7 (11.5)	77.3 (11.9)	< .001
< 55	1,198 (6.0%)	322 (4.5%)	753 (4.6%)	2,273 (5.3%)	< .001
55–84	13,126 (66.2%)	4,308 (60.8%)	10,696 (65.9%)	28,130 (65.2%)	
85 +	5,508 (27.8%)	2,451 (34.6%)	4,783 (29.5%)	12,742 (29.5%)	
**Sex**					
Female	11,951 (60.3%)	4,527 (63.9%)	9,913 (61.1%)	26,391 (61.2%)	< .001
**Falls Frequency (within the last 90 days)**					
0	12,753 (64.3%)	3,427 (48.4%)	8,662 (53.4%)	24,842 (57.6%)	< .001
1	4,306 (21.7%)	2,062 (29.1%)	4,530 (27.9%)	10,898 (25.3%)	
> 2	2,773 (14.0%)	1,592 (22.5%)	3,040 (18.7%)	7,405 (17.2%)	
**Diagnosis count**					
2	2,633 (13.3%)	923 (13.0%)	2,285 (14.1%)	5,841 (13.5%)	< .001
3	3,916 (19.7%)	1,331 (18.8%)	3,306 (20.4%)	8,553 (19.8%)	
4	4,158 (21.0%)	1,499 (21.2%)	3,479 (21.4%)	9,136 (21.2%)	
5 +	9,125 (46.0%)	3,328 (47.0%)	7,162 (44.1%)	19,615 (45.5%)	
**Ontario Marginalization Index**					
Low level of marginalization (Score = 1,2)	5,849 (29.5%)	2,150 (30.4%)	4,952 (30.5%)	12,951 (30.0%)	< .001
Medium level of marginalization (Score = 3)	8,454 (42.6%)	2,939 (41.5%)	6,768 (41.7%)	18,161 (42.1%)	
High level of marginalization (Score = 4,5)	5,345 (27.0%)	1,945 (27.5%)	4,434 (27.3%)	11,724 (27.2%)	
**ADL Hierarchy Scale**					
Independent	11,922 (60.1%)	3,562 (50.3%)	7,681 (47.3%)	23,165 (53.7%)	< .001
Supervision/limited Assistance	5,349 (27.0%)	2,043 (28.9%)	5,289 (32.6%)	12,681 (29.4%)	
Moderate/Extensive Assistance	2,561 (12.9%)	1,476 (20.8%)	3,262 (20.1%)	7,299 (16.9%)	
**IADL Involvement Scale**					
Independent/Set-up assist	650 (3.3%)	142 (2.0%)	191 (1.2%)	983 (2.3%)	< .001
Moderate/Extensive Assistance	19,182 (96.7%)	6,939 (98.0%)	16,041 (98.8%)	42,162 (97.7%)	
**CHESS Scale**					
No instability	2,418 (12.2%)	759 (10.7%)	1,207 (7.4%)	4,384 (10.2%)	< .001
Minimal instability	12,452 (62.8%)	4,690 (66.2%)	10,609 (65.4%)	27,751 (64.3%)	
Moderate/severe instability	4,962 (25.0%)	1,632 (23.0%)	4,416 (27.2%)	11,010 (25.5%)	
**Cognitive Performance Scale**					
No cognitive impairment	10,450 (52.7%)	2,965 (41.9%)	7,279 (44.8%)	20,694 (48.0%)	< .001
Mild cognitive impairment	6,796 (34.3%)	2,894 (40.9%)	6,545 (40.3%)	16,235 (37.6%)	
Moderate/Severe cognitive impairment	2,586 (13.0%)	1,222 (17.3%)	2,408 (14.8%)	6,216 (14.4%)	
**Depression Rating Scale**					
Score of 0 (no depressive symptoms)	11,532 (58.1%)	4,021 (56.8%)	9,075 (55.9%)	24,628 (57.1%)	0.003
Score of 1 or 2 (minimal symptoms present in last 3 days)	4,858 (24.5%)	1,766 (24.9%)	4,193 (25.8%)	10,817 (25.1%)	
Score of 3,4,5 (moderate number of symptoms in last 3 days)	2,421 (12.2%)	919 (13.0%)	2,105 (13.0%)	5,445 (12.6%)	
Score 6 + (severe/all mood symptoms present in last 3 days)	1,021 (5.1%)	375 (5.3%)	859 (5.3%)	2,255 (5.2%)	
**Pain Scale**					
No pain	6,898 (34.8%)	2,121 (30.0%)	5,130 (31.6%)	14,149 (32.8%)	< .001
Less than daily pain	1,968 (9.9%)	726 (10.3%)	1,619 (10.0%)	4,313 (10.0%)	
Daily pain	10,966 (55.3%)	4,234 (59.8%)	9,483 (58.4%)	24,683 (57.2%)	

Over a 3-month period, 10,100 individuals (23.4%) with multimorbidity who received home care services after transitioning home from acute care were readmitted to acute care (the hospital). Over a 12-month period, 18,218 (42.2%) were readmitted. At the 3-month follow-up time-frame, a higher proportion of individuals readmitted to the hospital were: 85 years of age and older (30.2%), had experienced two or more falls (18.6%), and had four or more chronic conditions (71.4%). A higher proportion of those readmitted required assistance with activities of daily living (52.2% vs 44.5%), experienced moderate/severe health instability (as captured through the CHESS scale) (35.5% vs 22.5%) and experienced some level of cognitive impairment (55.3% vs 51.1%) (*p* < 0.001) (Table 2). Similar proportions were observed at the 12-month follow-up across these demographics.

**Table 2:** Distribution of baseline characteristics and embedded health subscales stratified by hospital re-admission status

**Variable**	**3 months**			***P* value**	**12 Months**			***P* value**
	**Not readmitted *n* = 33,045 n(%)**	**Readmitted *n* = 10,100 n(%)**	**Total *N* = 43,145 n(%)**		**Not readmitted *n* = 24,927 n(%)**	**Readmitted *n* = 18,218 n(%)**	**Total *N* = 43,145 n(%)**	
**Home Care service group**								
Other home care services only	14,816 (44.8%)	5,016 (49.7%)	19,832 (46.0%)	< .001	10,989 (44.1%)	8,843 (48.5%)	19,832 (46.0%)	< .001
Rehabilitation therapy only	5,607 (17.0%)	1,474 (14.6%)	7,081 (16.4%)		4,293 (17.2%)	2,788 (15.3%)	7,081 (16.4%)	
Home care rehabilitation therapy and other services	12,622 (38.2%)	3,610 (35.7%)	16,232 (37.6%)		9,645 (38.7%)	6,587 (36.2%)	16,232 (37.6%)	
**Age**								
< 55	1,804 (5.5%)	469 (4.6%)	2,273 (5.3%)	0.002	1,451 (5.8%)	822 (4.5%)	2,273 (5.3%)	< .001
55–84	21,554 (65.2%)	6,576 (65.1%)	28,130 (65.2%)		16,466 (66.1%)	11,664 (64.0%)	28,130 (65.2%)	
85 +	9,687 (29.3%)	3,055 (30.2%)	12,742 (29.5%)		7,010 (28.1%)	5,732 (31.5%)	12,742 (29.5%)	
**Sex**								
Female	20,817 (63.0%)	5,574 (55.2%)	26,391 (61.2%)	< .001	16,064 (64.4%)	10,327 (56.7%)	26,391 (61.2%)	< .001
**Falls Frequency (within the last 90 days)**								
0	18,884 (57.1%)	5,958 (59.0%)	24,842 (57.6%)	< .001	14,296 (57.4%)	10,546 (57.9%)	24,842 (57.6%)	< .001
1	8,634 (26.1%)	2,264 (22.4%)	10,898 (25.3%)		6,559 (26.3%)	4,339 (23.8%)	10,898 (25.3%)	
2 +	5,527 (16.7%)	1,878 (18.6%)	7,405 (17.2%)		4,072 (16.3%)	3,333 (18.3%)	7,405 (17.2%)	
**Diagnosis count**								
2	4,689 (14.2%)	1,152 (11.4%)	5,841 (13.5%)	< .001	3,820 (15.3%)	2,021 (11.1%)	5,841 (13.5%)	< .001
3	6,813 (20.6%)	1,740 (17.2%)	8,553 (19.8%)		5,433 (21.8%)	3,120 (17.1%)	8,553 (19.8%)	
4	7,118 (21.5%)	2,018 (20.0%)	9,136 (21.2%)		5,479 (22.0%)	3,657 (20.1%)	9,136 (21.2%)	
5 +	14,425 (43.7%)	5,190 (51.4%)	19,615 (45.5%)		10,195 (40.9%)	9,420 (51.7%)	19,615 (45.5%)	
**Ontario Marginalization Index**								
Low level of marginalization (Score = 1,2)	9,947 (30.1%)	3,004 (29.7%)	12,951 (30.0%)	0.011	7,527 (30.2%)	5,424 (29.8%)	12,951 (30.0%)	0.033
Medium level of marginalization (Score = 3)	13,843 (41.9%)	4,318 (42.8%)	18,161 (42.1%)		10,469 (42.0%)	7,692 (42.2%)	18,161 (42.1%)	
High level of marginalization (Score = 4,5)	9,039 (27.4%)	2,685 (26.6%)	11,724 (27.2%)		6,777 (27.2%)	4,947 (27.2%)	11,724 (27.2%)	
**Marital Status**								
Never married	1,986 (6.0%)	532 (5.3%)	2,518 (5.8%)	< .001	1,534 (6.2%)	984 (5.4%)	2,518 (5.8%)	< .001
Married	14,537 (44.0%)	4,746 (47.0%)	19,283 (44.7%)		10,926 (43.8%)	8,357 (45.9%)	19,283 (44.7%)	
Divorced, separated, widowed	16,070 (48.6%)	4,696 (46.5%)	20,766 (48.1%)		12,111 (48.6%)	8,655 (47.5%)	20,766 (48.1%)	
Other	452 (1.4%)	126 (1.2%)	578 (1.3%)		356 (1.4%)	222 (1.2%)	578 (1.3%)	
**Bladder continence**								
Continent	21,364 (64.7%)	6,201 (61.4%)	27,565 (63.9%)	< .001	16,363 (65.6%)	11,202 (61.5%)	27,565 (63.9%)	< .001
Usually continent	6,518 (19.7%)	2,002 (19.8%)	8,520 (19.7%)		4,797 (19.2%)	3,723 (20.4%)	8,520 (19.7%)	
Usually incontinent	5,119 (15.5%)	1,872 (18.5%)	6,991 (16.2%)		3,737 (15.0%)	3,254 (17.9%)	6,991 (16.2%)	
**Bowel Continence**								
Continent	28,277 (85.6%)	8,052 (79.7%)	36,329 (84.2%)	< .001	21,410 (85.9%)	14,919 (81.9%)	36,329 (84.2%)	< .001
Usually continent	2,447 (7.4%)	994 (9.8%)	3,441 (8.0%)		1,783 (7.2%)	1,658 (9.1%)	3,441 (8.0%)	
Usually incontinent	2,288 (6.9%)	1,038 (10.3%)	3,326 (7.7%)		1,707 (6.8%)	1,619 (8.9%)	3,326 (7.7%)	
**ADL Hierarchy Scale**								
Independent	18,334 (55.5%)	4,831 (47.8%)	23,165 (53.7%)	< .001	13,928 (55.9%)	9,237 (50.7%)	23,165 (53.7%)	< .001
Supervision/limited Assistance	9,530 (28.8%)	3,151 (31.2%)	12,681 (29.4%)		7,106 (28.5%)	5,575 (30.6%)	12,681 (29.4%)	
Moderate/Extensive Assistance	5,181 (15.7%)	2,118 (21.0%)	7,299 (16.9%)		3,893 (15.6%)	3,406 (18.7%)	7,299 (16.9%)	
**IADL Involvement Scale**								
Independent/Set-up assist	772 (2.3%)	211 (2.1%)	983 (2.3%)	0.145	603 (2.4%)	380 (2.1%)	983 (2.3%)	0.022
Moderate/Extensive Assistance	32,273 (97.7%)	9,889 (97.9%)	42,162 (97.7%)		24,324 (97.6%)	17,838 (97.9%)	42,162 (97.7%)	
**CHESS Scale**								
No instability	3,638 (11.0%)	746 (7.4%)	4,384 (10.2%)	< .001	2,761 (11.1%)	1,623 (8.9%)	4,384 (10.2%)	< .001
Minimal instability	21,986 (66.5%)	5,765 (57.1%)	27,751 (64.3%)		16,764 (67.3%)	10,987 (60.3%)	27,751 (64.3%)	
Moderate/severe instability	7,421 (22.5%)	3,589 (35.5%)	11,010 (25.5%)		5,402 (21.7%)	5,608 (30.8%)	11,010 (25.5%)	
**Cognitive Performance Scale**								
No cognitive impairment	16,180 (49.0%)	4,514 (44.7%)	20,694 (48.0%)	< .001	12,542 (50.3%)	8,152 (44.7%)	20,694 (48.0%)	< .001
Mild cognitive impairment	12,310 (37.3%)	3,925 (38.9%)	16,235 (37.6%)		9,044 (36.3%)	7,191 (39.5%)	16,235 (37.6%)	
Moderate/Severe cognitive impairment	4,555 (13.8%)	1,661 (16.4%)	6,216 (14.4%)		3,341 (13.4%)	2,875 (15.8%)	6,216 (14.4%)	
**Depression Rating Scale**								
Score of 0 (no depressive symptoms)	19,272 (58.3%)	5,356 (53.0%)	24,628 (57.1%)	< .001	14,574 (58.5%)	10,054 (55.2%)	24,628 (57.1%)	< .001
Score of 1 or 2 (minimal symptoms present in last 3 days)	8,110 (24.5%)	2,707 (26.8%)	10,817 (25.1%)		6,131 (24.6%)	4,686 (25.7%)	10,817 (25.1%)	
Score of 3,4,5 (moderate number of symptoms in last 3 days)	4,034 (12.2%)	1,411 (14.0%)	5,445 (12.6%)		2,990 (12.0%)	2,455 (13.5%)	5,445 (12.6%)	
Score 6 + (severe/all mood symptoms present in last 3 days)	1,629 (4.9%)	626 (6.2%)	2,255 (5.2%)		1,232 (4.9%)	1,023 (5.6%)	2,255 (5.2%)	
**Pain Scale**								
No pain	10,711 (32.4%)	3,438 (34.0%)	14,149 (32.8%)	0.005	7,834 (31.4%)	6,315 (34.7%)	14,149 (32.8%)	< .001
Less than daily pain	3,291 (10.0%)	1,022 (10.1%)	4,313 (10.0%)		2,454 (9.8%)	1,859 (10.2%)	4,313 (10.0%)	
Daily pain	19,043 (57.6%)	5,640 (55.8%)	24,683 (57.2%)		14,639 (58.7%)	10,044 (55.1%)	24,683 (57.2%)	

When controlling for age, sex, and number of chronic conditions, those receiving rehabilitation therapy services only (occupational therapy and/or physical therapy) were less likely to be readmitted to the hospital (3-month: OR = 0.78; 95% CI = 0.73–0.83; 12-month: OR = 0.8; 95% CI = 0.76–0.85) than individuals who received other home care services. Those receiving a combination of rehabilitation therapy and other home care services were also less likely to be readmitted to the hospital (3-month: OR = 0.85; 95% CI = 0.81–0.89; 12-month: OR = 0.85; 95% CI = 0.82–0.89) compared to those receiving other home care services (Table 3).

**Table 3:** The association between receipt of home care rehabilitation therapy services and hospital readmission

**Outcome: 3-month readmission**						
	**Unadjusted Model**		Backwards Elimination Model^*^		**Model 2**^‡^	
	**Odds Ratio**	**95% Confidence Interval**	**Odds Ratio**	**95% Confidence Interval**	**Odds Ratio**	**95% Confidence Interval**
Other Home care services	Reference					
Home care rehabilitation therapy only	0.78	0.73–0.83	0.77	0.72–0.82	0.78	0.73–0.83
Home care rehabilitation therapy and other services	0.85	0.80–0.89	0.81	0.77–0.85	0.85	0.81–0.89
**Outcome: 12-month readmission**						
	**Unadjusted Model**		**Backwards Elimination Model**^†^		**Model 2**	
	**Odds Ratio**	**95% Confidence Interval**	**Odds Ratio**	**95% Confidence Interval**	**Odds Ratio**	**95% Confidence Interval**
Other Home care services	Reference					
Home care rehabilitation therapy only	0.81	0.76–0.85	0.79	0.75–0.84	0.8	0.76–0.85
Home care rehabilitation therapy and other services	0.85	0.81–0.89	0.82	0.79–0.86	0.85	0.82–0.89

^*^ Backwards elimination model covariates (outcome 3-month readmission): sex, ADL Scale, Pain Scale, CPS, DRS, CHESS, bowel continence, falls, and number of chronic conditions

^†^Backwards elimination model covariates (outcome 12-month readmission): sex, age, ADL, Pain Scale, CPS, DRS, CHESS, bowel incontinence, bladder incontinence, falls, number of chronic conditions

^‡^Model 2: age sex, number of chronic conditions

^§^ Each model contains covariates that align with the clinical characteristics component of the theoretical model discussed earlier; social characteristics were considered but not retained in the final models

Among the cohort, of individuals experiencing an emergency department visit(s) within the 3-month and 12-month period, a higher proportion experienced moderate/severe health instability (as measured by the CHESS) (3-month: 30.8% vs. 22.2%; 12-month: 27.5% vs. 22.0%), a higher proportion experienced moderate/severe cognitive impairment (3 month: 15.4% vs. 13.8%; 12-month: 14.8% vs. 13.7%), and five or more co-occurring chronic conditions (3-month: 49.6% vs. 42.9%; 12-month: 49.2% vs. 39.0%) (*p* < 0.001) (Table 4).

**Table 4:** Distribution of baseline characteristics and embedded health subscales stratified by emergency department use

**Variable**	**3 months**			***P* value**	**12 Months**			***P* value**
	**No emergency department use *n* = 26,603 n(%)**	**Emergency Department use *n* = 16,542 n(%)**	**Total *N* = 43,145 n(%)**		**No emergency department use *n* = 15,720 n(%)**	**Emergency department use *n* = 27,427 n(%)**	**Total *N* = 43,145 n(%)**	
**Home Care service group**								
Other home care services only	11,645 (43.8%)	8,187 (49.5%)	19,832 (46.0%)	< .001	6,652 (42.3%)	13,180 (48.1%)	19,832 (46.0%)	< .001
Rehabilitation therapy only	4,683 (17.6%)	2,398 (14.5%)	7,081 (16.4%)		2,762 (17.6%)	4,319 (15.7%)	7,081 (16.4%)	
Home care rehabilitation therapy and other services	10,275 (38.6%)	5,957 (36.0%)	16,232 (37.6%)		6,306 (40.1%)	9,926 (36.2%)	16,232 (37.6%)	
**Age**								
< 55	1,320 (5.0%)	953 (5.8%)	2,273 (5.3%)	0.001	796 (5.1%)	1,477 (5.4%)	2,273 (5.3%)	< .001
55–84	17,378 (65.3%)	10,752 (65.0%)	28,130 (65.2%)		10,443 (66.4%)	17,687 (64.5%)	28,130 (65.2%)	
85 +	7,905 (29.7%)	4,837 (29.2%)	12,742 (29.5%)		4,481 (28.5%)	8,261 (30.1%)	12,742 (29.5%)	
**Sex**								
Female	16,985 (63.8%)	9,406 (56.9%)	26,391 (61.2%)	< .001	10,221 (65.0%)	16,170 (59.0%)	26,391 (61.2%)	< .001
**Falls Frequency**								
0	15,131 (56.9%)	9,711 (58.7%)	24,842 (57.6%)	< .001	9,015 (57.3%)	15,827 (57.7%)	24,842 (57.6%)	< .001
1	7,031 (26.4%)	3,867 (23.4%)	10,898 (25.3%)		4,204 (26.7%)	6,694 (24.4%)	10,898 (25.3%)	
> 2	4,441 (16.7%)	2,964 (17.9%)	7,405 (17.2%)		2,501 (15.9%)	4,904 (17.9%)	7,405 (17.2%)	
**Diagnosis count**								
2	3,801 (14.3%)	2,040 (12.3%)	5,841 (13.5%)	< .001	2,513 (16.0%)	3,328 (12.1%)	5,841 (13.5%)	< .001
3	5,581 (21.0%)	2,972 (18.0%)	8,553 (19.8%)		3,554 (22.6%)	4,999 (18.2%)	8,553 (19.8%)	
4	5,814 (21.9%)	3,322 (20.1%)	9,136 (21.2%)		3,524 (22.4%)	5,612 (20.5%)	9,136 (21.2%)	
5 +	11,407 (42.9%)	8,208 (49.6%)	19,615 (45.5%)		6,129 (39.0%)	13,486 (49.2%)	19,615 (45.5%)	
**Ontario Marginalization Index**								
Low level of marginalization (Score = 1,2)	8,047 (30.2%)	4,904 (29.6%)	12,951 (30.0%)	< .001	4,784 (30.4%)	8,167 (29.8%)	12,951 (30.0%)	0.003
Medium level of marginalization (Score = 3)	11,047 (41.5%)	7,114 (43.0%)	18,161 (42.1%)		6,588 (41.9%)	11,573 (42.2%)	18,161 (42.1%)	
High level of marginalization (Score = 4,5)	7,342 (27.6%)	4,382 (26.5%)	11,724 (27.2%)		4,265 (27.1%)	7,459 (27.2%)	11,724 (27.2%)	
**Marital Status**								
Never married	1,555 (5.8%)	963 (5.8%)	2,518 (5.8%)	< .001	933 (5.9%)	1,585 (5.8%)	2,518 (5.8%)	0.01
Married	11,633 (43.7%)	7,650 (46.2%)	19,283 (44.7%)		6,858 (43.6%)	12,425 (45.3%)	19,283 (44.7%)	
Divorced, separated, widowed	13,070 (49.1%)	7,696 (46.5%)	20,766 (48.1%)		7,713 (49.1%)	13,053 (47.6%)	20,766 (48.1%)	
Other	345 (1.3%)	233 (1.4%)	578 (1.3%)					
**Bladder continence**								
Continent	17,060 (64.1%)	10,505 (63.5%)	27,565 (63.9%)	0.005	10,190 (64.8%)	17,375 (63.4%)	27,565 (63.9%)	0.009
Usually continent	5,319 (20.0%)	3,201 (19.4%)	8,520 (19.7%)		3,069 (19.5%)	5,451 (19.9%)	8,520 (19.7%)	
Usually incontinent	4,184 (15.7%)	2,807 (17.0%)	6,991 (16.2%)		2,438 (15.5%)	4,553 (16.6%)	6,991 (16.2%)	
**Bowel continence**								
Continent	22,771 (85.6%)	13,558 (82.0%)	36,329 (84.2%)	< .001	13,430 (85.4%)	22,899 (83.5%)	36,329 (84.2%)	< 0.001
Usually continent	1,964 (7.4%)	1,477 (8.9%)	3,441 (8.0%)		1,129 (7.2%)	2,312 (8.4%)	3,441 (8.0%)	
Usually incontinent	1,838 (6.9%)	1,488 (9.0%)	3,326 (7.7%)		1,138 (7.2%)	2,188 (8.0%)	3,326 (7.7%)	
	22,771 (85.6%)	13,558 (82.0%)	36,329 (84.2%)		13,430 (85.4%)	22,899 (83.5%)	36,329 (84.2%)	
**ADL Hierarchy Scale**								
Independent	14,744 (55.4%)	8,421 (50.9%)	23,165 (53.7%)	< .001	8,585 (54.6%)	14,580 (53.2%)	23,165 (53.7%)	0.013
Supervision/limited Assistance	7,691 (28.9%)	4,990 (30.2%)	12,681 (29.4%)		4,513 (28.7%)	8,168 (29.8%)	12,681 (29.4%)	
Moderate/Extensive Assistance	4,168 (15.7%)	3,131 (18.9%)	7,299 (16.9%)		2,622 (16.7%)	4,677 (17.1%)	7,299 (16.9%)	
**IADL Involvement Scale**								
Independent/Set-up assist	606 (2.3%)	377 (2.3%)	983 (2.3%)	0.994	340 (2.2%)	643 (2.3%)	983 (2.3%)	0.223
Moderate/Extensive Assistance	25,997 (97.7%)	16,165 (97.7%)	42,162 (97.7%)		15,380 (97.8%)	26,782 (97.7%)	42,162 (97.7%)	
**CHESS Scale**								
No instability	2,941 (11.1%)	1,443 (8.7%)	4,384 (10.2%)	< .001	1,679 (10.7%)	2,705 (9.9%)	4,384 (10.2%)	< .001
Minimal instability	17,754 (66.7%)	9,997 (60.4%)	27,751 (64.3%)		10,582 (67.3%)	17,169 (62.6%)	27,751 (64.3%)	
Moderate/severe instability	5,908 (22.2%)	5,102 (30.8%)	11,010 (25.5%)		3,459 (22.0%)	7,551 (27.5%)	11,010 (25.5%)	
**Cognitive Performance Scale**								
No cognitive impairment	13,091 (49.2%)	7,603 (46.0%)	20,694 (48.0%)	< .001	8,005 (50.9%)	12,689 (46.3%)	20,694 (48.0%)	< .001
Mild cognitive impairment	9,843 (37.0%)	6,392 (38.6%)	16,235 (37.6%)		5,558 (35.4%)	10,677 (38.9%)	16,235 (37.6%)	
Moderate/Severe cognitive impairment	3,669 (13.8%)	2,547 (15.4%)	6,216 (14.4%)		2,157 (13.7%)	4,059 (14.8%)	6,216 (14.4%)	
**Depression Rating Scale**								
Score of 0 (no depressive symptoms)	15,756 (59.2%)	8,872 (53.6%)	24,628 (57.1%)	< .001	9,423 (59.9%)	15,205 (55.4%)	24,628 (57.1%)	< .001
Score of 1 or 2 (minimal symptoms present in last 3 days)	6,449 (24.2%)	4,368 (26.4%)	10,817 (25.1%)		3,758 (23.9%)	7,059 (25.7%)	10,817 (25.1%)	
Score of 3,4,5 (moderate number of symptoms in last 3 days)	3,143 (11.8%)	2,302 (13.9%)	5,445 (12.6%)		1,810 (11.5%)	3,635 (13.3%)	5,445 (12.6%)	
Score 6 + (severe/all mood symptoms present in last 3 days)	1,255 (4.7%)	1,000 (6.0%)	2,255 (5.2%)		729 (4.6%)	1,526 (5.6%)	2,255 (5.2%)	
**Pain Scale**								
No pain	8,666 (32.6%)	5,483 (33.1%)	14,149 (32.8%)	0.328	4,963 (31.6%)	9,186 (33.5%)	14,149 (32.8%)	< .001
Less than daily pain	2,643 (9.9%)	1,670 (10.1%)	4,313 (10.0%)		1,535 (9.8%)	2,778 (10.1%)	4,313 (10.0%)	
Daily pain	15,294 (57.5%)	9,389 (56.8%)	24,683 (57.2%)		9,222 (58.7%)	15,461 (56.4%)	24,683 (57.2%)	

When controlling for age, sex, and number of chronic conditions, in comparison to people receiving other home care services, those receiving rehabilitation therapy services only (occupational therapy and/or physical therapy) were less likely to use emergency department services (3-month: OR = 0.73; 95% CI = 0.69–0.78; 12-month: OR = 0.79; 95% CI = 0.75–0.83). Compared to other home care services, clients receiving a combination of home care rehabilitation therapy and other home care services were also less likely to use emergency department services (3-month: OR = 0.83; 95% CI = 0.80–0.87; 12-month: 0.8; 95% CI = 0.77–0.84) (Table 5). When controlling for these three covariates in the model examining the 3-month emergency department use outcome, the overall fit of the model was inadequate, however, the magnitude of the association between receipt of rehabilitation services and subsequent hospital utilization was the same in the unadjusted model and the backwards elimination model. The relationship between receipt of rehabilitation services was therefore still considered clinically relevant. These models were examined for multicollinearity, and it was not present.

**Table 5:** The association between receipt of rehabilitation therapy services and emergency department use

**Outcome**: **3-month emergency department use**						
	**Unadjusted Model**		Backwards Elimination Model^*^		**Model 2**^‡^	
	**Odds Ratio**	**95% Confidence Interval**	**Odds Ratio**	**95% Confidence Interval**	**Odds Ratio**	**95% Confidence Interval**
Other Home care services	Reference					
Home care rehabilitation therapy only	0.73	0.69–0.77	0.73	0.69–0.78	0.73	0.69–0.78
Home care rehabilitation therapy and other services	0.83	0.79–0.86	0.81	0.77–0.84	0.83	0.8–0.87
**Outcome: 12-month emergency department use**						
	**Unadjusted Model**		**Backwards Elimination Model**^†^		**Model 2**	
	**Odds Ratio**	**95% Confidence Interval**	**Odds Ratio**	**95% Confidence Interval**	**Odds Ratio**	**95% Confidence Interval**
Other Home care services	Reference					
Home care rehabilitation therapy only	0.79	0.75–0.84	0.78	0.74–0.83	0.79	0.75–0.83
Home care rehabilitation therapy and other services	0.79	0.76–0.83	0.79	0.75–0.82	0.8	0.77–0.84

^*^ Backwards elimination model covariates (outcome 3-month emergency department use): age, sex, ADL Scale, DRS, CHESS, bladder continence, bowel continence, falls, Ontario marginalization summary score, and number of chronic conditions

^†^Backwards elimination model covariates (outcome 12-month emergency department use): sex, age, CPS, DRS, CHESS, bowel continence, falls, pain, number of chronic conditions

^‡^Model 2-age, sex, and number of chronic conditions

^§^ models contain covariates that align with the clinical and social characteristics components of the theoretical model discussed earlier

The secondary analysis evaluated the association between receipt of rehabilitation therapy and the number of hospital admissions and emergency department visits (counts) within the 3-month and 12-month windows. During this time, a similar health utilization trend was observed for the therapy services. When controlling for age, sex, and number of chronic conditions, those who received rehabilitation therapy only were less likely to be admitted to the hospital (3-month Rate Ratio = 0.73; 95% CI = 0.68–0.78; 12-month Rate Ratio = 0.79; 95% CI = 0.75–0.83) and less likely to utilize emergency department services (3-month Rate Ratio = 0.69; 95% CI = 0.66–0.73; 12-month Rate Ratio = 0.79; 95% CI = 0.76–0.82) compared to those receiving other home care services only (Table 6).

**Table 6:** The association between home care rehabilitation therapy and healthcare utilization

**Hospital Readmission**				
	**3 Months**		**12 months**	
	**Rate Ratio**	**95% Confidence Interval**	**Rate Ratio**	**95% Confidence Interval**
Other Home care services	Reference			
Home care rehabilitation therapy only	0.73	0.68- 0.78	0.79	0.75-0.83
Home care rehabilitation therapy and other services	0.82	0.78- 0.87	0.86	0.83–0.89
**Emergency Department Use**				
	**3 Months**		**12 months**	
	**Rate Ratio**	**95% Confidence Interval**	**Rate Ratio**	**95% Confidence Interval**
Other Home care services	Reference			
Home care rehabilitation therapy only	0.69	0.66 -0.73	0.79	0.76 -0.82
Home care rehabilitation therapy and other services	0.82	0.79 -0.86	0.84	0.82 -0.87

*• healthcare utilization as a count variable-negative binomial regression*

^*^ adjusted for: age, sex, and number of chronic conditions

## Discussion

In this study, we examined the relationship between the receipt of long-stay home care rehabilitation therapy and hospital readmission and emergency department use following an acute hospital discharge among persons with multimorbidity. This study offers an important contribution to the literature and suggests home care rehabilitation therapy is associated with lower hospital readmission and emergency department use in people with multimorbidity after an acute hospital discharge.

We found that persons receiving home care services, irrespective of the type of services, were more likely to require at least moderate assistance with instrumental activities of daily living, were older, and had a similar number of chronic conditions that they experienced. This finding is consistent with results across existing population-based home care studies, and highlights the important role home care services play in supporting older adults to live within their community [40, 41].

The profile of home care clients that received rehabilitation therapy in comparison to people that received other home care services may indicate that service referrals were made congruently with the therapists’ area of expertise. For example, the literature has consistently shown that people receiving rehabilitation therapy services tend to require higher levels of support with activities of daily living, have experienced fall(s), and experience cognitive impairment [42, 43]. In the current study, a higher proportion of home care clients receiving rehabilitation therapy experienced functional impairment, suggesting that home care rehabilitation therapists are providing services to this group, and the areas of functional decline are consistent with existing home care rehabilitation literature among other populations.

Our findings revealed that individuals receiving rehabilitation therapy services, whether alone or with other home care services, were less likely to be re-admitted to the hospital and less likely to use the emergency department services compared to those receiving other home care services only. This is consistent with the literature that has found home care rehabilitation was associated with a reduction in unplanned healthcare use by people who have experienced a stroke, older adults, and patients with musculoskeletal health conditions, and adds to the growing evidence highlighting the potential value of home care rehabilitation in reducing future unplanned healthcare use [11, 44–46]. One study explored the relationship between receipt of home care rehabilitation and health care utilization among older adults in a small geographic region within Ontario [44]. The authors found that people receiving physical therapy had the longest length of time before being re-hospitalized [44]. The current study considered multimorbidity across a broader chronic health condition profile that considered a range of 17 chronic conditions across cognitive, cardiorespiratory, and psychoemotional domains of health. The findings of the current study suggest rehabilitation therapists may help reduce subsequent healthcare utilization amongst a group of medically complex clients and their role can be leveraged to support hospital to home care transitions.

A recent observational study found that increased spending on hospital-based occupational therapy was the only healthcare service that reduced hospital readmissions among patients with a diagnosis of pneumonia, acute myocardial infarction, or heart failure [10]. The authors found that increased spending on occupational therapy in hospital lowered 30-day hospital readmissions. The authors hypothesized that this may be because occupational therapists focused on the immediate functional and social needs of the patients [10]. A recent study by Freburger et al. [47], revealed that receipt of acute inpatient rehabilitation services during an acute hospital admission for individuals with pneumonia or influenza was associated with reductions in hospital readmissions. The authors found that the inverse relationship between receipt of therapy services and 30-day hospital readmissions was stronger as the number of therapy visits increased. Specifically, only statistically significant reductions were observed among the group that received 6 + therapy visits (OR = 0.86; 95%CI:0.75–0.98) [47]. Another study examined the association between receipt of inpatient occupational therapy services, and the frequency and intensity of these services on 30-day readmission rates for individuals diagnosed with common cardiorespiratory conditions, and those requiring joint replacements [48]. Edelstein et al. [48] also found that those receiving a higher frequency of acute care occupational therapy services were 1% less likely to be readmitted; however, these results should be interpreted with caution as the 95% confidence intervals ranged from 0.99–1.00. Similarly, a systematic review identifying interventions aimed at promoting early hospital discharge and preventing hospital (re)admissions found that interventions delivered in the home were associated with reduced hospital length of stay and improved patient satisfaction; however, these were not rehabilitation specific [49]. Our results build on these findings by suggesting that rehabilitation therapy delivered in the home also reduces hospital readmissions and emergency department visits for individuals with multimorbidity. The results of the current study also highlight the need for further investigation into the types and duration of interventions delivered by trained rehabilitation therapists.

The findings of the current study add to the growing body of literature that demonstrates the value of rehabilitation therapy services in reducing unplanned hospital admissions and emergency department use [44–46, 50–53]. As summarized above, there is evidence in the literature across varied populations that occupational therapy and/or physical therapy aid in successful transitions to home with durable discharges from acute care facilities [10, 11, 45]. In this context, the term ‘durable discharge(s)’ is used to describe a successful and sustained transition from the hospital setting to home. Ontario health care is undergoing significant reform and is moving towards an integrated model of care delivery whereby coordinated services are easily navigated by both the patient and the provider [54]. Therefore, our results suggest health system planners should consider facilitating increased use of home care rehabilitation therapy as a means of reducing unplanned hospitalization and emergency department visits for people with multimorbidity who are transitioning home after an acute hospital stay. As the health system continues this transition, there will be potential to utilize integrated system-level data across health care sectors to further investigate the impact of rehabilitation on health care utilization outcomes during a period of policy change.

Another potential area of future research that would extend the findings of this study would be to conduct an economic analysis to investigate the cost–benefit of expenditures on rehabilitation therapy for the health system. The findings from this study shed light on the association between home care rehabilitation therapy and healthcare utilization after one of the most common health care transitions (hospital to home). Future research could use the findings from this study as the foundation for examining the relationship between home care rehabilitation and healthcare costs. Additionally, the relationship between receipt of rehabilitation therapy and other healthcare outcomes could be examined such as discharges to long-term care, discharges from home care services, functional changes, and mortality [11].

## Limitations

The selection of chronic conditions chosen for inclusion was limited to 17 and there is the possibility that some people with multimorbidity were not captured in this cohort. This selection does however consider the chronic conditions with the heaviest healthcare burden and is consistent with other ICES literature that utilized similar data [7, 14, 15, 17–19, 55–57]. Inconsistencies exist with respect to defining multimorbidity within the growing body of multimorbidity literature. Inconsistent definitions of the term ‘multimorbidity’ creates a significant barrier for comparisons at both the micro- and macro-levels. It was therefore important for the authors to maintain a consistent definition within the ICES data for two reasons: 1) it works towards contributing to the growing body of multimorbidity literature in a consistent way that can be compared to previous literature, and 2) it helps build and establish a consistent definition for future research.

This study also only considers long-stay home care clients. Short-stay clients were excluded from this study because full RAI-HC assessments are not completed for those on a short-stay caseload following an acute change in medical status. Long-stay home care clients align with the population of interest, those with multiple chronic conditions, because of the chronic nature of their diagnosis and prolonged health care interaction. Cook et al. [11], highlighted that long-stay home care clients are not often referred to home care rehabilitation and as such, this study may underestimate rehabilitation referrals and the association between receipt of rehabilitation therapy and health care utilization. This presents an opportunity for future research to consider the inclusion of short-stay home care clients as a means of capturing a more comprehensive representation of home care rehabilitation users. Another direction for future research in this area could be stratification by whether receipt of rehabilitation was a new service or a re-instatement of existing rehabilitation services. Future studies could also consider longitudinal analysis of interRAI data to capture competing interests such as alternative discharges from home care including long-term care admissions or deaths; similar to the work conducted by Cook et al. [11]

Additionally, this study was limited by the variables collected across health administrative databases and therefore, there may be the potential for unmeasured and uncontrolled confounding. Data were also limited to what is captured within existing datasets. One particular area of data sparsity related to the receipt of rehabilitation services within the RAI-HC was with respect to the frequency, intensity, and type of therapeutic intervention. There is information related to the cumulative number of days, hours, and minutes of home care services that were provided in the previous week or since last assessment if it had been conducted less than seven days prior; however, there are gaps in data collection that extend beyond the previous week of services. Similarly, there were gaps with respect to receipt of rehabilitation therapy delivered within the acute care hospital setting. This information does not provide a comprehensive picture of the cumulative rehabilitation services delivered. Understanding receipt of home care services as a whole, may require further investigation into the services provided prior to discharge as well as between RAI-HC assessments that are beyond the seven-day timeframe that the assessment provides. Furthermore, understanding of supports that extend beyond the home care funded services, such as region-specific programming, consideration of the recently developed caregiver risk evaluation algorithm, duration of rehabilitation services, and system-level covariates, could be next steps in this research. Examination of these covariates may also provide further explanation of the enduring results related to receipt of rehabilitation observed at the 12-month mark in the current study.

## Conclusions

This study took a population-level approach to understanding the demographics of those with multimorbidity receiving home care rehabilitation therapy after acute care hospitalization, and the association between receipt of home care rehabilitation therapy and subsequent health care utilization. We found that there was an inverse relationship between receipt of home care rehabilitation and hospital admissions and emergency department visits over 3-month and 12-month periods following discharge from an acute care hospital. This work provides a platform to further examine rehabilitation specific interventions among those with multimorbidity and economic value of rehabilitation therapies, both in times of healthcare reform and health care stability.

## Data Availability

The dataset from this study is held securely in coded form at ICES. While legal data sharing agreements between ICES and data providers (e.g., healthcare organizations and government) prohibit ICES from making the dataset publicly available, access may be granted to those who meet pre-specified criteria for confidential access, available at www.ices.on.ca/DAS (email: das@ices.on.ca). The full dataset creation plan and underlying analytic code are available from the authors upon request, understanding that the computer programs may rely upon coding templates or macros that are unique to ICES and are therefore either inaccessible or may require modification.

## References

[1] Nguyen H, Manolova G, Daskalopoulou C, Vitoratou S, Prince M, Prina AM (2019). Prevalence of multimorbidity in community settings: a systematic review and meta-analysis of observational studies. J Comorbidity.

[2] Payne RA, Abel GA, Guthrie B, Mercer SW (2013). The effect of physical multimorbidity, mental health conditions and socioeconomic deprivation on unplanned admissions to hospital: a retrospective cohort study. CMAJ.

[3] Condelius A, Edberg AK, Jakobsson U, Hallberg IR (2008). Hospital admissions among people 65+ related to multimorbidity, municipal and outpatient care. Arch Gerontol Geriatr.

[4] Friedman B, Jiang HJ, Elixhauser A, Segal A (2006). Hospital inpatient costs for adults with multiple chronic conditions. Med Care Res Rev.

[5] Marengoni A, Angleman S, Melis R, Mangialasche F, Karp A, Garmen A (2011). Aging with multimorbidity: a systematic review of the literature. Ageing Res Rev.

[6] Wolff JL, Starfield B, Anderson G (2002). Prevalence, expenditures, and complications of multiple chronic conditions in the elderly. Arch Intern Med.

[7] Mondor L, Maxwell CJ, Bronskill SE, Gruneir A, Wodchis WP (2016). The relative impact of chronic conditions and multimorbidity on health-related quality of life in Ontario long-stay home care clients. Qual Life Res.

[8] World Health Organization (2016). Multimorbidity: Technical series on safer primary care.

[9] World Health Organization. Towards a Common Language for Functioning, Disability and Health: ICF [Internet]. Geneva, Switzerland; 2002 [cited 2021 June 10]. Available from: http://www.who.int/classifications/icf/training/icfbeginnersguide.pdf

[10] Rogers AT, Bai G, Lavin RA, Anderson GF (2016). Higher hospital spending on occupational therapy is associated with lower readmission rates. Med Care Res Rev.

[11] Cook RJ, Berg K, Lee K, Poss JW, Hirdes JP, Stolee P (2013). Rehabilitation in home care is associated with functional improvement and preferred discharge. Arch Phys Med Rehabil.

[12] Mofina A, Miller J, Tranmer J, Donnelly C (2020). Home care rehabilitation therapy services for individuals with multimorbidity: a rapid review. J Comorbidity.

[13] Gentry K, Snyder K, Barstow B, Hamson-Utley J. The Biopsychosocial Model: Application to Occupational Therapy Practice. Open J Occup Ther. 2018;6(4).

[14] Koné Pefoyo AJ, Bronskill SE, Gruneir A, Calzavara A, Thavorn K, Petrosyan Y (2015). The increasing burden and complexity of multimorbidity disease epidemiology - Chronic. BMC Public Health.

[15] Gruneir A, Bronskill SE, Maxwell CJ, Bai YQ, Kone AJ, Thavorn K (2016). The association between multimorbidity and hospitalization is modified by individual demographics and physician continuity of care: a retrospective cohort study. BMC Health Serv Res.

[16] Mondor L, Cohen D, Khan AI, Wodchis WP (2018). Income inequalities in multimorbidity prevalence in Ontario, Canada: a decomposition analysis of linked survey and health administrative data. Int J Equity Health.

[17] Mondor L, Maxwell CJ, Hogan DB, Bronskill SE, Gruneir A, Lane NE (2017). Multimorbidity and healthcare utilization among home care clients with dementia in Ontario, Canada: a retrospective analysis of a population-based cohort. PLoS Med.

[18] Lane NE, Maxwell CJ, Gruneir A, Bronskill SE, Wodchis WP (2015). Absence of a socioeconomic gradient in older adults’ survival with multiple chronic conditions. EBioMedicine.

[19] Thavorn K, Maxwell CJ, Gruneir A, Bronskill SE, Bai Y, Koné Pefoyo AJ (2017). Effect of socio-demographic factors on the association between multimorbidity and healthcare costs: a population-based, retrospective cohort study. BMJ Open.

[20] Rosella L, Kornas K, Huang A, Bornbaum C, Henry D, Wodchis WP (2018). Accumulation of chronic conditions at the time of death increased in Ontario from 1994 to 2013. Health Aff.

[21] Petrosyan Y, Bai YQ, Koné Pefoyo AJ, Gruneir A, Thavorn K, Maxwell CJ (2017). The relationship between diabetes care quality and diabetes-related hospitalizations and the modifying role of comorbidity. Can J Diabetes.

[22] Tu K, Campbell NR, Chen Z-L, Cauch-Dudek KJ, McAlister FA (2007). Accuracy of administrative databases in identifying patients with hypertension. Open Med.

[23] Gershon AS, Wang C, Guan J, Vasilevska-Ristovska J, Cicutto L, To T (2009). Identifying individuals with physcian diagnosed COPD in health administrative databases. COPD.

[24] Gershon AS, Wang C, Guan J, Vasilevska-Ristovska J, Cicutto L, To T (2009). Identifying patients with physician-diagnosed asthma in health administrative databases. Can Respir J.

[25] Hux JE, Ivis F, Flintoft V, Bica A (2002). Determination of prevalence and incidence using a validated administrative algorithm. Diabetes Care.

[26] Schultz SE, Rothwell DM, Chen Z, Tu K (2013). Identifying cases of congestive heart failure from administrative data: a validation study using primary care patient records. Chronic Dis Inj Can.

[27] Austin PC, Daly PA, Tu JV (2002). A multicenter study of the coding accuracy of hospital discharge administrative data for patients admitted to cardiac care units in Ontario. Am Heart J.

[28] Landi F, Tua E, Onder G, Carrara B, Sgadari A, Rinaldi C, et al. Minimum data set for home care : a valid instrument to assess frail older people living in the community. Med Care. 2000;38(12):1184–90. 10.1097/00005650-200012000-00005 11186297

[29] Hirdes JP, Ljunggren G, Morris JN, Frijters DH, Finne Soveri H, Gray L (2008). Reliability of the interRAI suite of assessment instruments: a 12-country study of an integrated health information system. BMC Health Serv Res.

[30] Hirdes JP, Frijters DH, Teare GF (2003). The MDS-CHESS scale: a new measure to predict mortality in institutionalized older people. J Am Geriatr Soc.

[31] Burrows AB, Morris JN, Simon SE, Hirdes JP, Phillips C (2000). Development of a minimum data set-based depression rating scale for use in nursing homes. Age Ageing.

[32] Morris JN, Fries BE, Morris SA (1999). Scaling ADLs within the MDS. J Gerontol A Biol Sci Med Sci.

[33] The government of Ontario. Home and Community Care Support Services: Frequently Asked Questions [Internet]. 2021 [cited 2021 Aug 1]. Available from: http://healthcareathome.ca/hnhb/en/Getting-Care/Patient-and-Caregiver-Resources/Frequently-Asked-Questions

[34] The government of Ontario. Home and community care [Internet]. 2021 [cited 2021 Aug 1]. Available from: https://www.ontario.ca/page/homecare-seniors

[35] Fusco D, Bochicchio GB, Onder G, Barillaro C, Bernabei R, Landi F (2009). Predictors of rehabilitation outcome among frail elderly patients living in the community. J Am Med Dir Assoc.

[36] Armstrong JJ, Zhu M, Hirdes JP, Stolee P (2015). Rehabilitation therapies for older clients of the Ontario home care system: regional variation and client-level predictors of service provision. Disabil Rehabil.

[37] Mofina AM, Guthrie DM (2014). A comparison of home care quality indicator rates in two Canadian provinces. BMC Health Serv Res.

[38] Byers AL, Allore H, Gill TM, Peduzzi PN (2003). Application of negative binomial modeling for discrete outcomes: a case study in aging research. J Clin Epidemiol.

[39] Hutchinson MK, Holtman MC (2005). Analysis of count data using poisson regression. Res Nurs Heal.

[40] Campitelli MA, Bronskill SE, Hogan DB, Diong C, Amuah JE, Gill S (2016). The prevalence and health consequences of frailty in a population-based older home care cohort: a comparison of different measures. BMC Geriatr.

[41] Jones A, Bronskill SE, Agarwal G, Seow H, Feeny D, Costa AP (2019). The primary care and other health system use of home care patients: a retrospective cohort analysis. CMAJ Open.

[42] Rehabilitative Care Alliance. Community-Based Rehabilitation : Providing High-Value Rehabilitative Care in the Community [Internet]. Ontario, Canada; 2020 [cited 10 August 2021]. Available from: http://www.rehabcarealliance.ca/uploads/File/Initiatives_and_Toolkits/Community_Rehab/RCA_Community-based_Rehab_White_Paper_Part_2.pdf

[43] Canadian Home Care Assocation. Rehabilitation therapy services in home care: Evidence [Internet]. 2011. [cited 2021 May 20] Available from: https://books.scholarsportal.info/en/read?id=/ ebooks/ebooks0/gibson_cppc- chrc/2012–05–31/1/10545174

[44] Knott TC. Home Based Rehabilitation and its Impact on Hospital Utilization [dissteration on the Internet]. Queen’s University, 2013 [cited 2019 Feb 21] Available from: https://qspace.library.queensu.ca/bitstream/handle/1974/8092/Knott_T_Christine_finalsubmission201306_PhD.pdf?sequence=1&isAllowed=y

[45] Langhorne P, Taylor G, Murray G, Dennis M, Anderson C, Bautz-Holter E (2005). Early supported discharge services for stroke patients: a meta-analysis of individual patients’ data. Lancet.

[46] Stolee P, Lim SN, Wilson L, Glenny C. Inpatient versus home-based rehabilitation for older adults with musculoskeletal disorders : a systematic review. Clin Rehabil. 2012;26(5):387–402. 10.1177/0269215511423279 21971753

[47] Freburger JK, Chou A, Euloth T, Matcho B (2020). Variation in acute care rehabilitation and 30-day hospital readmission or mortality in adult patients with pneumonia. JAMA Netw Open.

[48] Edelstein J, Walker R, Middleton A, Reistetter T, Gary KW, Reynolds S (2022). Higher frequency of acute occupational therapy services is associated with reduced hospital readmissions. Am J Occup Ther.

[49] Coffey A, Leahy-Warren P, Savage E, Hegarty J, Cornally N, Day MR (2019). Interventions to promote early discharge and avoid inappropriate hospital (Re)admission: a systematic review. Int J Environ Res Public Health.

[50] Barnes MP, Radermacher H (2001). Neurological rehabilitation in the community. J Rehabil Med.

[51] Langhorne P, Bernhardt J, Kwakkel G (2011). Stroke rehabilitation. Lancet.

[52] Langhorne P, Baylan S (2017). Early supported discharge services for people with acute stroke: a cochrane review summary. Cochrane Database Syst Rev.

[53] Catty J, Burns T, Knapp M, Watt H, Wright C, Henderson J (2002). Home treatment for mental health problems: a systematic review. Psychol Med.

[54] Ministry of Health and Long-Term Care. Ontario Health Teams : Guidance for Health Care Providers and Organizations [Internet]. Ontario, Canada; 2019 [cited 2021 Apr 10]. Available from: https://health.gov.on.ca/en/pro/programs/connectedcare/oht/docs/guidance_doc_en.pdf

[55] Public Health Agency of Canada. Economic burden of illness in Canada 2005–2008 [Internet]. 2014 [cited 2021 Apr 20]. Available from: http://www.phac-aspc.gc.ca/publicat/ebic-femc98/pdf/ebic1998.pdf

[56] Canadian Institute for Health Information. Seniors and the Health Care System : What Is the Impact of Multiple Chronic Conditions ? [Internet]. Canada; 2011 [cited 2021Apr 21]. Available from: https://secure.cihi.ca/free_products/air-chronic_disease_aib_en.pdf

[57] Public Health Agency of Canada. Report on the State of Canada Public Health in Canada 2010, Growing older-Adding life to years [Internet]. Canada; 2010 [cited 2021 Apr 21]. Available from: http://www.phac-aspc.gc.ca/cphorsphc-respcacsp/2010/fr-rc/pdf/cpho_report_2010_e.pdf

